# The complete mitochondrial genome of the starfish *Coscinasterias acutispina* Stimpson, 1862 (Echinodermata, Forcipulatida) from the East China Sea

**DOI:** 10.1080/23802359.2022.2134748

**Published:** 2022-10-21

**Authors:** Kangkang Han, Xiaoqi Zeng, Gang Ni

**Affiliations:** a Ministry of Education, Key Laboratory of Mariculture, Ocean University of China, Qingdao, China; b Institute of Evolution and Marine Biodiversity, Ocean University of China, Qingdao, China

**Keywords:** Echinodermata, Forcipulatida, complete mitogenome, phylogenetic analysis

## Abstract

In this study, we determined the first complete mitochondrial genome sequence of *Coscinasterias acutispina* Stimpson, 1862. The mitogenome was 16,186 bp in length and contained 13 protein-coding genes, two ribosomal RNA genes, and 22 transfer RNA genes. The gene order and direction were identical to those of other asteroid starfish species. Phylogenetic analysis showed that *C. acutispina* was located at the basal position of Forcipulatida, belonging to the genus *Coscinasterias* within the family of Asteriidae.

The asteroid starfish *Coscinasterias acutispina* Stimpson, 1862, widely distributed in the shallow waters of the northwestern Pacific (Huang 1994), represents an important benthonic organism that can affect marine macroecology. Former studies of this species have focused on its morphological characteristics, reproductive biology, and population genetics (e.g. Haramoto et al. 2006; Shibata et al. 2011; Seto et al. 2013). Phylogenetic study on this species, however, is still insufficient considering only morphological features and partial gene fragments are available (Waters and Roy 2003). Here, to clarify the mitochondrial genome characteristics of *C. acutispina* and its phylogenetic position within Forcipulatida, we sequenced and analyzed its mitochondrial genome sequence.

An adult specimen of *C. acutispina* was collected from the Nanji Island, Zhejiang Province, China (27°28′18″N, 121°2′7″E) in September 2021 and deposited in the Fisheries Biology Research Laboratory at Ocean University of China (https://scxy.ouc.edu.cn/main.htm, contact person: Xiaoqi Zeng, email: zengxq@ouc.edu.cn) under the voucher no. ZJ-NJ2109018. Since *C. acutispina* is neither an endangered nor protected species in China, no specific permissions or licenses are needed for collection. Total mitochondrial DNA was extracted from tube feet using DNA Extraction Kit (TSINGKE, Beijing, China) according to the manufacturer’s instructions. The genomic DNA library was constructed using TruSeq DNA Sample Preparation Guide. The whole mitochondrial genome was sequenced using a next-generation sequencing platform (Illumina HiSeq X Ten, PE150, San Diego, CA). Raw data were trimmed using trimmomatic v0.38 (Bolger et al. 2014). Clean sequences were assembled using SPAdes v3.10.1 and NOVOPlasty software with default parameters (Dierckxsens et al. 2017). The PCGs, rRNAs, and tRNAs were annotated using MITOS web server with echinoderm/flatworm mitochondrial genetic codes (Bernt et al. 2013). Phylogenetic analysis was performed based on the sequences of 13 protein-coding genes (PCGs) using maximum-likelihood (ML) with IQ-TREE (Nguyen et al. 2015). ModelFinder was used to select the best-fit partition model and partition schemes (Kalyaanamoorthy et al. 2017). Nodal support was assessed with 5000 ultrafast bootstrap replicates (Minh et al. 2013).

The complete mitochondrial genome of *C. acutispina* (GenBank accession no. OM272844) was a circular DNA molecule with 16,186 bp in size, which contained 13 PCGs, two rRNAs, and 22 tRNAs. The overall nucleotide composition of *C. acutispina* showed obvious AT bias, with a high AT content of 62.6% (A: 31.9%; T: 30.7%; G: 13.8%; C 23.6%). The gene order and transcription direction were identical to those of other asteroid starfish species. Most PCGs were initiated by typical ATG codon as start codon, while ND2 and ND1 were initiated with GTG codon and ND4L with ATT. The 12 PCGs were terminated with complete termination codon (TAA or TAG), except for CytB gene with an incomplete termination codon T.

A phylogenetic tree was constructed based on the 13 PCG sequences of 23 Asteroidea mitochondrial genomes using ML method (ML tree, Figure 1), with two echinoids as outgroups (*Echinocardium cordatum*, GenBank accession no. FN562581; *Strongylocentrotus pallidus*, AM900392). The ML tree showed that *C. acutispina* formed a monophyletic cade with high nodal support value, and then formed a large clade with *Asterias amurensis* (AB183559), *Distolasterias nipon* (MH473231), *Aphelasterias japonica* (KM244709), and *Pisaster ochraceus* (NC_042741). Those results provided valuable information for further investigation of the phylogeny of Asteroidea.

**Figure 1.**
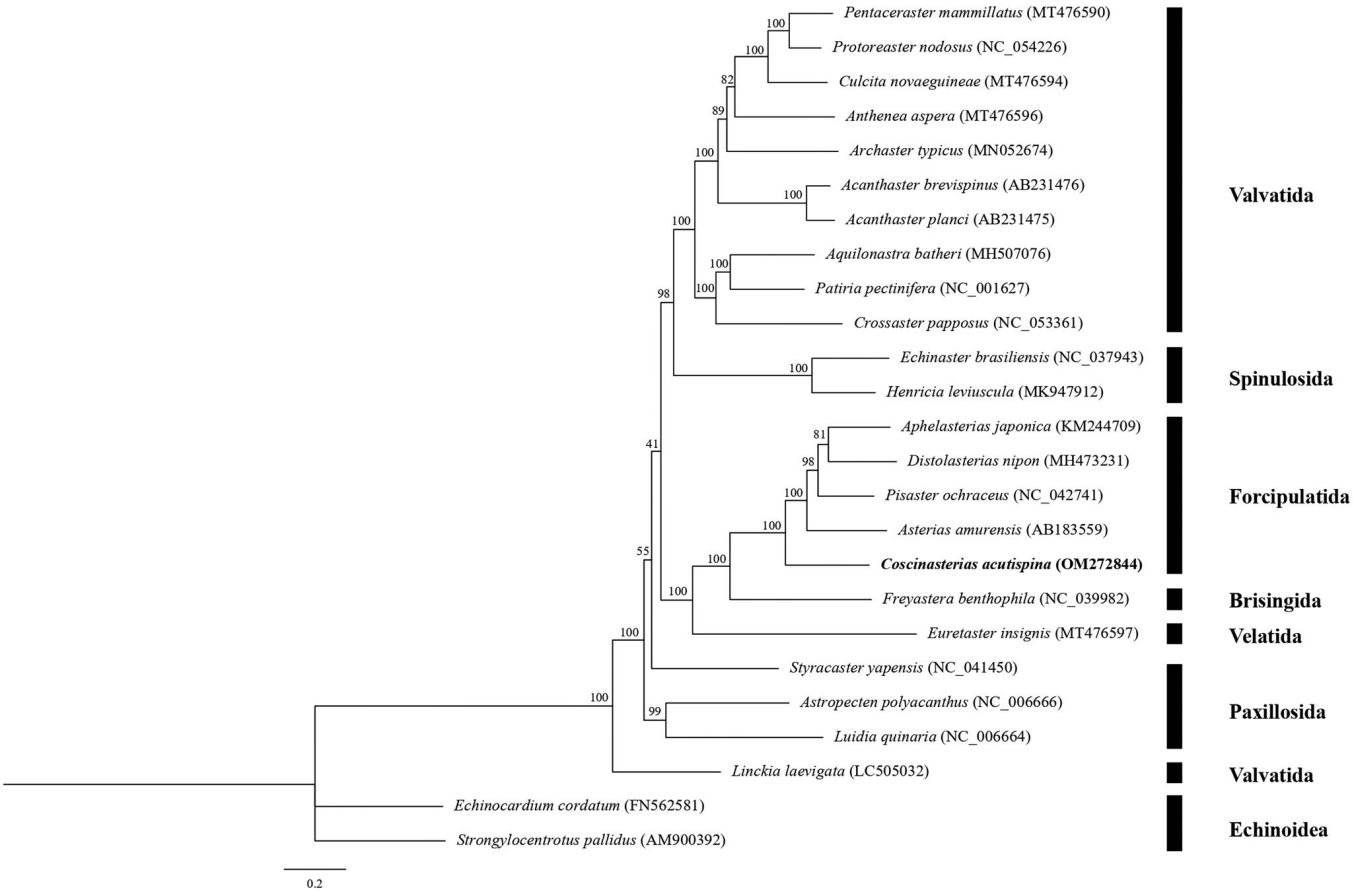
Maximum-likelihood (ML) tree based on the nucleotide sequences of 13 PCGs of 23 asteroid mitochondrial genomes with two echinoids as outgroups.

## Data Availability

The genome sequence that supports the findings of this study was openly available in GenBank of NCBI under accession no. OM272844 (https://www.ncbi.nlm.nih.gov/nuccore/OM272844.1/). BioProject no.: PRJNA833891; BioSample no.: SAMN28021061; SRA no.: SRR19025778.

## References

[CIT0001] Bernt M, Donath A, Jühling F, Externbrink F, Florentz C, Fritzsch G, Pütz J, Middendorf M, Stadler PF. 2013. MITOS: improved de novo metazoan mitochondrial genome annotation. Mol Phylogenet Evol. 69(2):313–319. 22982435 10.1016/j.ympev.2012.08.023

[CIT0002] Bolger AM, Lohse M, Usadel B. 2014. Trimmomatic: a flexible trimmer for Illumina sequence data. Bioinformatics. 30(15):2114–2120. 24695404 10.1093/bioinformatics/btu170 PMC4103590

[CIT0003] Dierckxsens N, Mardulyn P, Smits G. 2017. NOVOPlasty: de novo assembly of organelle genomes from whole genome data. Nucleic Acids Res. 45(4):e18. 28204566 10.1093/nar/gkw955 PMC5389512

[CIT0004] Haramoto S, Komatsu M, Yamazaki Y. 2006. Population genetic structures of the fissiparous seastar *Coscinasterias acutispina* in the Sea of Japan. Mar Biol. 149(4):813–820.

[CIT0005] Huang ZG. 1994. Marine species and their distributions in China’s seas. Beijing: China Ocean Press.

[CIT0006] Kalyaanamoorthy S, Bui Quang M, Wong TKF, von Haeseler A, Jermiin LS. 2017. ModelFinder: fast model selection for accurate phylogenetic estimates. Nat Methods. 14(6):587–589. 28481363 10.1038/nmeth.4285 PMC5453245

[CIT0007] Minh BQ, Nguyen MAT, von Haeseler A. 2013. Ultrafast approximation for phylogenetic bootstrap. Mol Biol Evol. 30(5):1188–1195. 23418397 10.1093/molbev/mst024 PMC3670741

[CIT0008] Nguyen L-T, Schmidt HA, Von Haeseler A, Minh BQ. 2015. IQ-TREE: a fast and effective stochastic algorithm for estimating maximum-likelihood phylogenies. Mol Biol Evol. 32(1):268–274. 25371430 10.1093/molbev/msu300 PMC4271533

[CIT0009] Seto Y, Komatsu M, Wakabayashi K, Fujita D. 2013. Asexual reproduction of *Coscinasterias acutispina* (Stimpson, 1862) in tank culture. Cahiers Biol Mar. 54:641–647.

[CIT0010] Shibata D, Hirano Y, Komatsu M. 2011. Life cycle of the multiarmed sea star *Coscinasterias acutispina* (Stimpson, 1862) in laboratory culture: sexual and asexual reproductive pathways. Zoolog Sci. 28(5):313–317. 21557653 10.2108/zsj.28.313

[CIT0011] Waters J, Roy M. 2003. Global phylogeography of the fissiparous sea-star genus *Coscinasterias*. Mar Biol. 142(1):185–191.

